# Mirabegron versus vibegron in previously untreated female patients with overactive bladder: A randomized, single‐clinic, open‐label trial

**DOI:** 10.1111/luts.12480

**Published:** 2023-05-04

**Authors:** Hirotaka Sato, Shota Otsuka, Sachiyuki Tsukada

**Affiliations:** ^1^ Department of Urology Hokusuikai Kinen Hospital Ibaraki Japan; ^2^ Department of Orthopedic Surgery Hokusuikai Kinen Hospital Ibaraki Japan

**Keywords:** female, mirabegron, overactive bladder, overactive bladder symptom score, quality of life, vibegron

## Abstract

**Objectives:**

This study aimed to assess the efficacy and safety of mirabegron compared with vibegron (both 50 mg once daily) in Japanese female patients with symptoms of overactive bladder (OAB).

**Methods:**

This prospective, 12‐week, two‐arm, parallel‐group, open‐label randomized trial (UMIN000038288) was conducted at a single clinic from December 2019 to September 2022. The primary efficacy outcome measure was the change in mean total overactive bladder symptom scores (OABSSs) from baseline to end of treatment (EOT) (Week 12). The secondary efficacy outcome measures were changes in mean International Prostate Symptom Score from baseline to EOT, the ratio of patients who achieved a minimal clinically important change (MCIC) of total OABSS, and individual domains of the King's Health Questionnaire. Safety assessments, such as adverse events (AEs), postvoid residual volume, and patient‐reported incidences, were recorded at every visit.

**Results:**

There was no statistically significant adjusted mean difference between mirabegron and vibegron in terms of the primary outcome of the mean change from baseline to EOT in the total OABSS. The difference in the percentage of patients in the mirabegron and vibegron groups achieving an MCIC on the total OABSS was not statistically significant but appeared to be clinically important. The incidence of treatment‐related AEs was significantly higher for the vibegron group (38.5%) than the mirabegron group (19.1%) (*p* = .047).

**Conclusions:**

These results showed that both drugs were effective in female OAB patients, with no significant differences in terms of efficacy. However, the safety of vibegron requires further investigation.

## 1 INTRODUCTION

Overactive bladder (OAB) syndrome is characterized by urinary urgency, with or without urgency incontinence, generally accompanied by increased daytime frequency and nocturia in the absence of an obvious condition such as urinary tract infection (UTI).^1^ The prevalence of OAB increases with age,^2^ and a Japanese survey estimated that 8.1 million adults (12.4%) over the age of 40 years are affected by OAB in Japan.^3^ In this study, 37% of participants aged 80 years and older had OAB, compared to only 5% of those aged 40–49 years.^3^ The population of Japan is aging at an unprecedented rate, with the percentage of elderly people in Japan exceeding that in all other countries^4^; in 2021, 28.9% of the Japanese population was 65 years or older, while 14.9% were 75 years or older.^4^ Furthermore, OAB is known to have a significant negative impact on quality of life (QoL)^5^, ^6^ in elderly individuals and is associated with increased risk of falls,^7^ fractures with the use of anticholinergics,^8^ depression,^9^ and sleep disturbances.^10^, ^11^

Anticholinergics and selective β_3_‐adrenergic receptor (AR) agonists are the primary drug therapies used to treat OAB symptoms in Japan^12^; however, they can result in important adverse events (AEs) caused by systemic anticholinergic effects,^13^ and there have been reports of poor medication persistence.^14^, ^15^ There is also emerging evidence to support an association between cumulative anticholinergic use and the risk of cognitive impairment and dementia, with a recent study reporting an increased risk of dementia (odds ratio [OR]: 1.19–1.65) with using bladder anticholinergics.^16^

Another OAB drug target, the β_3_‐AR agonist, was developed as an alternative oral therapy and was approved for marketing in Japan in 2011.^17^ Vibegron, approved for use in Japan in 2018, is a novel, potent β_3_‐AR agonist that is highly selective for the β_3_ receptor in vitro.^18^ In a recent phase III study conducted in Japan, vibegron demonstrated sustained efficacy versus placebo for all OAB symptoms.^19^

Herein, we conducted a prospectively designed randomized controlled trial (RCT) on the safety and efficacy of mirabegron and vibegron in routine clinical settings. This is the first novel study in Japan to examine the efficacy and safety of β_3_ agonists in untreated elderly women aged over 70 years with OAB. Further, this is the first head‐to‐head RCT to report the efficacy and safety of mirabegron and vibegron simultaneously.

## 2 METHODS

### 2.1 Design

This study was designed as a prospective trial involving postmenopausal female patients in a single clinic between December 2019 and September 2022. A randomized, open‐label, parallel‐group trial design (registration number UMIN000038288) was adopted to compare the safety and efficacy of mirabegron and vibegron. The total study duration was 12 weeks, encompassing a 12‐week treatment period. This study was performed in accordance with the Declaration of Helsinki and was approved by the institutional review board of our institute (2019–037). Written informed consent was obtained from all participants. Postmenopausal treatment‐naïve female OAB patients were included in this study. OAB was diagnosed using the overactive bladder symptom score (OABSS) validated by Homma et al.^3^ based on the OABSS definition (a score ≥2 points for Question 3 [urgency] of the OABSS combined with a total score ≥3 points). The OABSS is a four‐item questionnaire used to evaluate daytime frequency, nocturia, urgency, and urinary urgency incontinence (UUI) in the past week. Among patients with OAB, those with a UUI score ≥2 were defined as OAB wet,^10^, ^20^ and all others were defined as OAB dry. A mean change from baseline to end of treatment (EOT) of 3 or more points on the total OABSS was defined as a minimal clinically important change (MCIC).^21^ MCIC was further defined as a mean change from baseline to EOT of 5 points on King's Health Questionnaire (KHQ)^22^ domains.^23^ The KHQ is a worldwide questionnaire for health‐related QoL in patients with OAB.

### 2.2 Participants

Patients scheduled for the OAB trial were invited by one of the treating physicians (H.S.) to participate in this study between December 2019 and September 2022. Eligible patients were treatment‐naïve postmenopausal females with OAB. The exclusion criteria were premenopausal women, previous pharmacotherapy for OAB, contraindications for the use of β_3_‐AR agonists, acute UTI, pregnancy, severe heart failure, predominant stress incontinence, uncontrolled diabetes or hypertension, severe renal or hepatic impairment, obvious neurogenic bladder, postvoid residual urine volume (PVR) >150 mL, and patients who were judged ineligible by the attending physicians.

#### 2.2.1 Randomization

Patients were randomly allocated to the two treatment groups in a 1:1 ratio. Block randomization, performed by an independent investigator not involved in the actual treatment, was used to generate a random‐number sequence by permuted block randomization with a block size of 4 using computer software R (R Foundation for Statistical Computing, Vienna, Austria). Investigators and patients were not blinded to the group allocations.

### 2.3 Procedure

Patients were randomized to receive oral mirabegron (50 mg) or vibegron (50 mg) once daily for 12 weeks. The 12‐week study consisted of a pretreatment period (Visit 1, baseline), a 4‐week treatment period (Visit 2), an 8‐week treatment period (Visit 3), and a 12‐week treatment period (Visit 4). The examined efficacy outcomes were the changes in the following items from baseline to 4, 8, and 12 weeks after treatment: OABSS, OABSS domain, International Prostate Symptom Score (IPSS), IPSS voiding symptoms (IPSS‐V), IPSS storage symptoms (IPSS‐S), IPSS QoL score, PVR, and KHQ score.

### 2.4 Outcomes

The primary efficacy outcome measure was the change in the mean number of OABSSs from baseline to the EOT (Week 12). The secondary efficacy outcome measures were the baseline‐to‐EOT changes in the mean number of OABSS domains (daytime frequency, nocturia, urgency, and UUI) and the mean number of IPSS‐S, IPSS‐V, and IPSS‐QoL; the ratio of patients who achieved an MCIC in OABSS; and individual domains of the KHQ, as well as the percentage of dry and wet OAB at EOT for the two groups.

The safety assessment was conducted based on AEs, and PVR was performed at Visits 1 (baseline), 2 (Week 4), 3 (Week 8), and 4 (Week 12). Vital signs (systolic blood pressure [SBP], diastolic blood pressure [DBP], and heart rate [HR]) were checked at Visits 1 and 4. Treatment‐related adverse events (TRAEs) were defined as any drug reactions related to the treatment agents.

### 2.5 Sample size

No statistical sample‐size calculations were conducted. However, a sample size of 44 patients per group resulted in a post hoc power of 24% to detect differences in the mean of 0.7 points, assuming a common SD of 3.0, based on a type I error rate of 5%.

Safety analysis was performed on patients in the safety analysis set (SAF), and efficacy analysis was performed primarily on the full analysis set (FAS). The SAF comprised randomized patients who received one dose of the study drug and underwent a safety measurement. The FAS included SAF patients with at least one efficacy measurement after the initial treatment. The QOL analysis set was defined as FAS for patients for whom at least one domain score could be calculated and who had taken the study drug for at least 28 days.

### 2.6 Statistical analysis

The least squares mean and two‐sided 95% CIs of changes in the primary and secondary efficacy outcome measures from baseline to EOT in the mirabegron and vibegron groups were calculated using an analysis of covariance (ANCOVA) model adjusting for the treatment group, age, and baseline value. The percentage change in OABSS and KHQ score from baseline to EOT achieving MCIC and the percentage of OAB dry and wet at EOT were calculated using a logistic regression model including the same factors as the ANCOVA models, with ORs and 95% CIs comparing the treatment effects between the mirabegron and vibegron groups. Multiple imputations were used for all missing data.

All tests were two‐sided, and significance was set at *p* < .05. All statistical analyses were performed using R and EZR (Saitama Medical Center, Jichi Medical University, Saitama, Japan) software.

## 3 RESULTS

**FIGURE 1:**
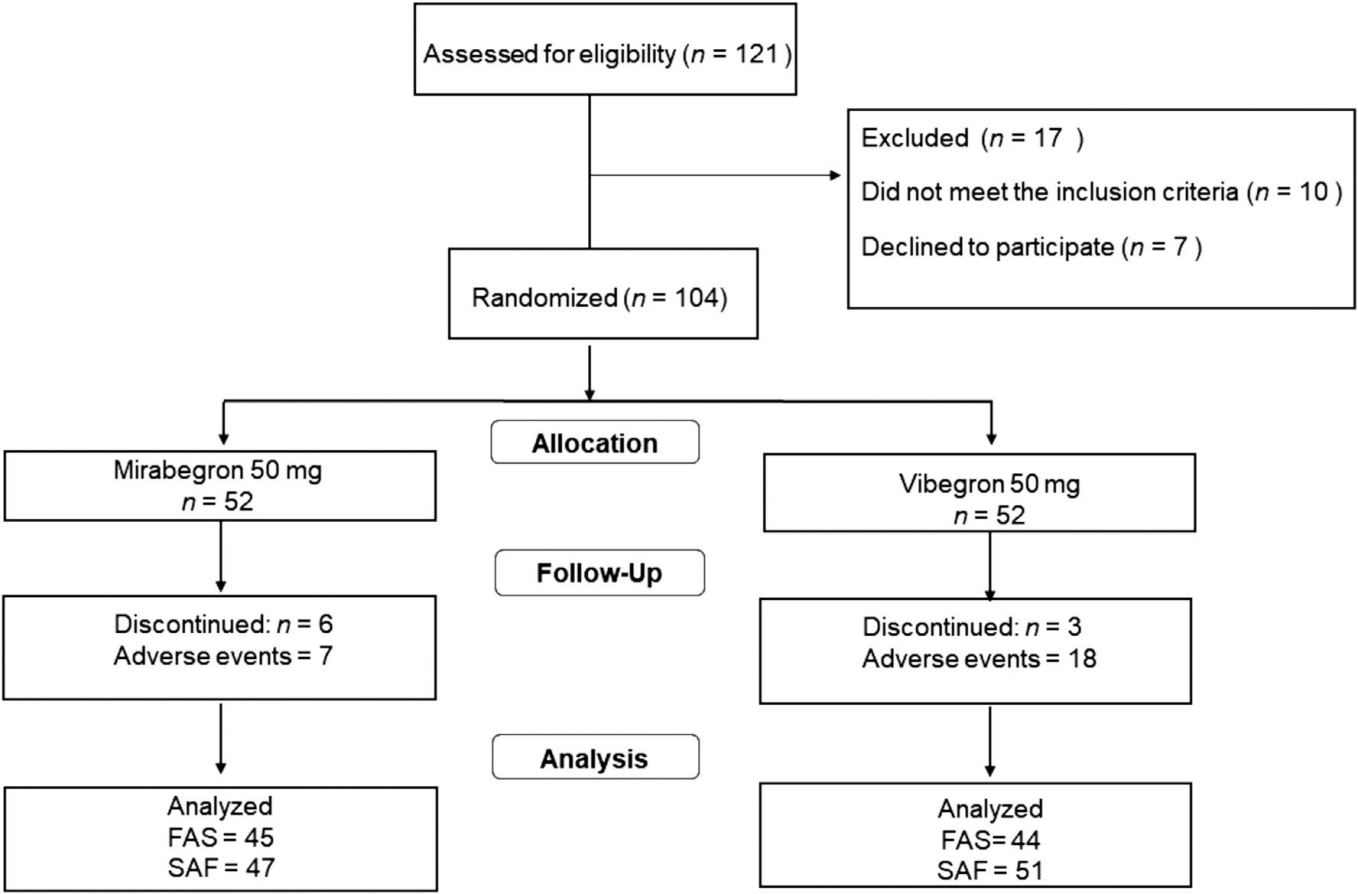
Patient disposition. FAS, full analysis set; SAF, safety analysis set.

A total of 104 patients were randomized to the two treatment groups (50 mg mirabegron, *n* = 52; 50 mg vibegron, *n* = 52), and 9 of the 104 patients withdrew during the treatment period (mirabegron, *n* = 6; vibegron, *n* = 3) (Figure 1). Demographic and baseline characteristics in the FAS population (*n* = 89) were similar between the two treatment groups, with no statistically significant differences, except for the proportion of diabetes patients within each group (mirabegron vs. vibegron, 12/45 [26.7%] vs. 3/44 [6.8%]) (Table 1). The etiology of OAB was idiopathic in all 89 patients. Patients with two or more complex comorbidities (hypertension, diabetes, stroke, angina pectoris, and osteoporosis) accounted for 18% of the mirabegron group and 14% of the vibegron group.

**TABLE 1:** Baseline demographic and OAB characteristics (full analysis) of the study participants.

Demographic characteristics	Mirabegron, 50 mg (*n* = 45)	Vibegron, 50 mg (*n* = 44)
Age, (years)		
Mean (SD)	74 (9.2)	73 (8.2)
<65, *n* (%)	7 (16)	7 (16)
≥65, *n* (%)	38 (84)	37 (84)
BMI (kg/m^2^)		
Mean (SD)	24 (3.6)	24 (3.6)
Parity (SD)	2.1 (0.9)	2.1 (0.9)
Diabetes, *n* (%)	12 (27)	3 (6.8)
Type of OAB		
OAB wet, *n* (%)	10 (27)	14 (37)
OAB dry, *n* (%)	27 (73)	24 (63)
OABSS ± SD		
Total score	8.8 ± 3.1	9.3 ± 2.6
Q1. Daytime frequency	0.8 ± 0.5	1.1 ± 0.6
Q2. Nocturia	2.0 ± 1.0	2.1 ± 1.0
Q3. Urgency	3.2 ± 1.2	3.3 ± 1.2
Q4. UUI	2.8 ± 1.3	3.0 ± 1.5
IPSS		
Total score	12 ± 6.2	11 ± 7.9
Voiding	3.4 ± 3.6	2.4 ± 3.8
Storage	7.0 ± 3.5	7.0 ± 3.8
QOL score	4.9 ± 1.4	5.2 ± 0.9
KHQ		
General health perceptions	45 ± 19.6	41 ± 20.8
Incontinence impact	57 ± 29.4	57 ± 30.5
Role limitations	33 ± 21.6	44 ± 28.9
Physical limitations	36 ± 25.5	43 ± 30.0
Social limitations	20 ± 21.5	29 ± 29.8
Personal relationship	4.0 ± 9.6	7.9 ± 21.8
Emotions	47 ± 26.9	51 ± 31.1
Sleep and energy	38 ± 29.0	39 ± 26.3
Symptom severity	42 ± 19.9	43 ± 23.5

*Note*: Data are shown as numbers (%) or mean ± SD.

Abbreviations: BMI, body mass index; IPSS, International Prostate Symptom Score; KHQ, King's Health Questionnaire; OAB, overactive bladder; OABSS, overactive bladder symptom score; QoL, quality of life; UUI, urgency urinary incontinence.

**FIGURE 2:**
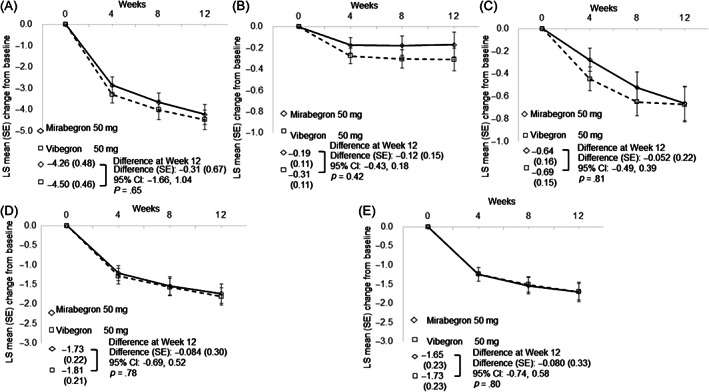
Changes in LS mean (SE) from baseline to 12 weeks in overactive bladder symptom score (OABSS). (A) Total OABSS; (B) daytime frequency; (C) nighttime frequency; (D) urgency; and (E) urgency urinary incontinence. I‐bar indicates SE. LS mean, least squares mean.

**FIGURE 3:**
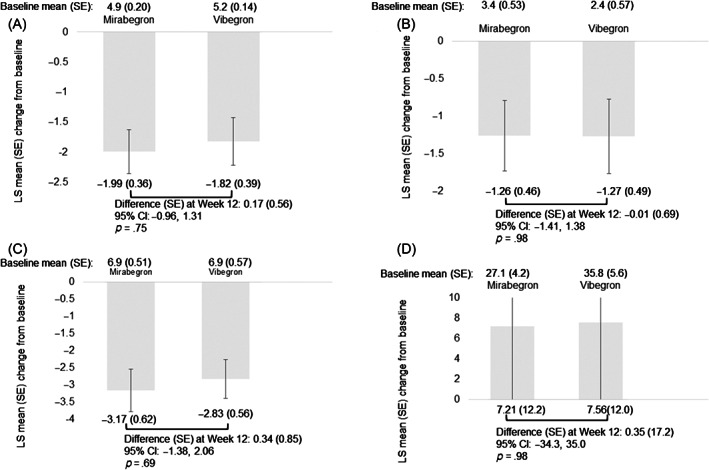
Changes in LS mean (SE) from baseline to 12 weeks in (A) International Prostate Symptom Score quality of life (IPSS‐QoL); (B) IPSS voiding symptoms; (C) IPSS storage symptoms; and (D) postvoid residual urine volume. I‐bar indicates SE. LS mean, least squares mean; QoL, quality of life.

Regarding the primary outcome, there was no statistically significant adjusted mean difference in the mean change in OABSS from baseline to EOT between the mirabegron and vibegron groups (difference [SE]: −0.31 [0.67]; 95% CI: −1.66, 1.04) (Figure 2A; Table 2). For the secondary outcomes, the mean change in difference from baseline to EOT in the OABSS domains (daytime frequency, nighttime frequency, urgency, and UUI) is shown in Figure 2 and Table 2. No statistically significant adjusted mean change in daytime frequency scores from baseline to EOT was observed between the mirabegron and vibegron groups (difference [SE]: −0.12 [0.15]; 95% CI: −0.43, 0.18), mean number of nocturia scores (difference [SE]: −0.052 [0.22]; 95% CI: −0.49, 0.39), mean change in urgency score (difference [SE]: −0.084 [0.30]; 95% CI: −0.69, 0.52), or mean change in UUI score (difference [SE]: −0.080 [0.33]; 95% CI: −0.74, 0.58) (Figure 2B–E, respectively; Table 2). No statistically significant adjusted mean change in IPSS QoL from baseline to EOT was observed in either group (difference [SE]: 0.17 [0.56]; 95% CI: −0.96, 1.31), mean change in IPSS‐V (difference [SE]: −0.01 [0.69]; 95% CI: −1.41, 1.38), or mean change in IPSS‐S (difference [SE]: 0.34 [0.85]; 95% CI: −1.38, 2.06) (Figure 3A–C, respectively; Table 2).

**TABLE 2:** Efficacy results at Week 12 compared to baseline (full analysis set).

Mirabegron				Vibegron			
	LS mean	SE	95% CI	LS mean	SE	95% CI	*p*
OABSS 4 weeks	−2.85	0.382	−3.61 to −2.09	−3.29	0.386	−4.05 to −2.52	.428
OABSS 8 weeks	−3.77	0.412	−4.59 to −2.95	−4.11	0.401	−4.90 to −3.31	.562
OABSS 12 weeks	−4.26	0.476	−5.21 to −3.32	−4.50	0.462	−5.42 to −3.58	.717
Q1 4 weeks	−0.175	0.0727	−0.319 to −0.030	−0.276	0.0737	−0.423 to −0.130	.3327
Q1 8 weeks	−0.177	0.091	−0.358 to −0.0042	−0.304	0.0877	−0.479 to −0.130	.335
Q1 12 weeks	−0.169	0.116	−0.401 to 0.062	−0.310	0.107	−0.523 to −0.097	.4018
Q2 4 weeks	−0.276	0.102	−0.478 to −0.0746	−0.445	0.103	−0.649 to −0.2408	.247
Q2 8 weeks	−0.521	0.138	−0.795 to −0.247	−0.649	0.121	−0.890 to −0.408	.487
Q2 12 weeks	−0.661	0.152	−0.964 to −0.359	−0.672	0.154	−0.979 to −0.365	.962
Q3 4 weeks	−1.21	0.189	−1.58 to −0.830	−1.29	0.192	−1.67 to −0.908	.761
Q3 8 weeks	−1.54	0.229	−1.99 to −1.08	−1.57	0.217	−2.00 to −1.14	.918
Q3 12 weeks	−1.73	0.247	−2.22 to −1.24	−1.81	0.22	−2.25 to −1.37	.806
Q4 4 weeks	−1.24	0.176	−1.59 to −0.886	−1.24	0.178	−1.59 to −0.884	.991
Q4 8 weeks	−1.54	0.222	−1.98 to −1.09	−1.51	0.203	−1.91 to −1.10	.924
Q4 12 weeks	−1.70	0.251	−2.19 to −1.20	−1.70	0.218	−2.14 to −1.27	.983
IPSS QoL	−1.99	0.368	−2.72 to −1.26	−1.82	0.397	−2.61 to −1.03	.754
IPSS voiding	−1.26	0.467	−2.19 to −0.330	−1.27	0.493	−2.25 to −0.292	.984
IPSS storage	−3.17	0.620	−4.41 to −1.94	−2.83	0.563	−3.95 to −1.71	.693
Change_PVR	7.21	12.2	−17.1 to 31.5	7.56	12.0	−16.2 to 31.4	.983

	Estimated OR	SE	95% CI	*p*
MCIC of OABSS (vibegron against mirabegron)	2.30	0.54	0.782 to 6.766	.127
OAB dry	0.696	0.501	0.255 to 1.894	.472
General health perceptions	0.342	0.509	0.123 to 0.947	.039*
Incontinence impact	1.084	0.469	0.425 to 2.765	.863
Role limitations	1.430	0.467	0.563 to 3.630	.445
Physical limitations	1.201	0.474	0.466 to 3.094	.699
Social limitations	1.169	0.470	0.457 to 2.989	.740
Personal relationships	0.641	0.660	0.169 to 2.423	.505
Emotions	0.753	0.503	0.276 to 2.057	.576
Sleep/energy	0.768	0.454	0.310 to 1.901	.564
Severity	0.495	0.533	0.170 to 1.437	.192

*Note*: Only domains in general health perceptions are provided with an asterisk due to statistically significant differences.

Abbreviations: IPSS, International Prostate Symptom Score; LS mean, least squares mean; MCIC, minimal clinically important change; OAB, overactive bladder; OABSS, overactive bladder symptom score; OR, odds ratio; PVR, postvoid residual urine volume; QoL, quality of life.

**FIGURE 4:**
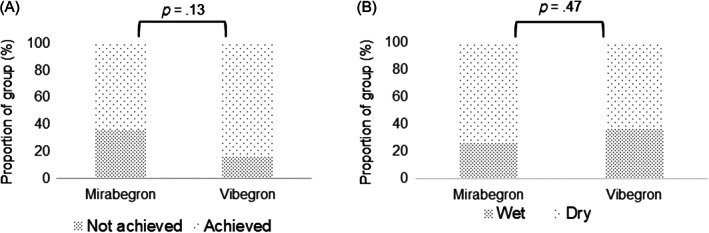
(A) Percentage change in MCIC of OABSS from baseline to end of treatment (Week 12) achieving MCIC. (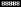) Not achieved, 35.1% and 15.8%; (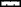) achieved, 64.9% and 84.2%, for mirabegron and vibegron, respectively. (B) Proportion of groups with dry and wet OAB at Week 12. (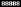) OAB wet, 27.0% and 36.8% (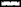); OAB dry, 73.0% and 63.2%, for mirabegron and vibegron, respectively. MCIC was defined as reduction of ≥3 points in total score. OAB wet was defined as score of ≥2 points on OABSS Question 4; otherwise, OAB dry was defined. MCIC, minimal clinically important change; OAB, overactive bladder; OABSS, overactive bladder symptom score.

**FIGURE 5:**
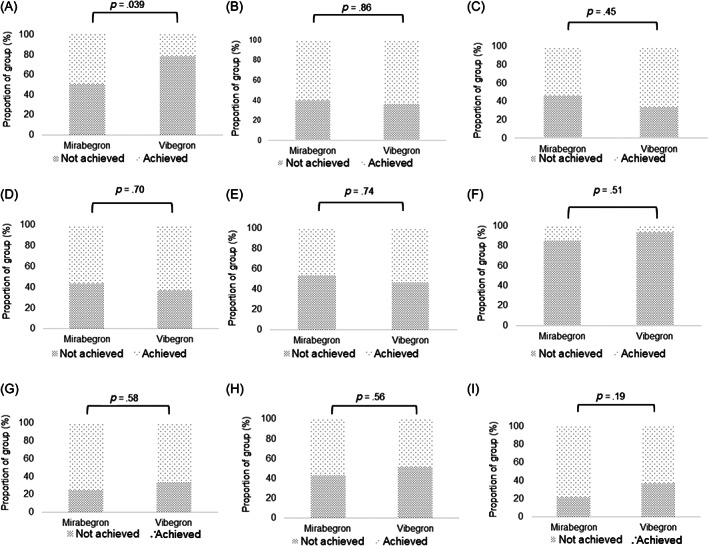
Percentage of change in KHQ from baseline to Week 12 in those achieving MCIC. (A) General health perceptions: (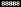) not achieved, 51.4% and 78.9%; (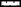) achieved, 48.6% and 21.1%, for mirabegron and vibegron, respectively. (B) Incontinence impact: not achieved, 40.5% and 36.8%; achieved, 59.5% and 63.2%, for mirabegron and vibegron, respectively. (C) Role limitations: not achieved, 45.9% and 34.2%; achieved, 54.1% and 65.8%, for mirabegron and vibegron, respectively. (D) Physical limitations: not achieved, 43.2% and 36.8%; achieved, 56.8% and 63.2%, for mirabegron and vibegron, respectively. (E) Social limitations: not achieved, 54.1% and 47.4%; achieved, 45.9% and 52.6%, for mirabegron and vibegron, respectively. (F) Personal relationships: not achieved, 85.2% and 93.3%; achieved, 14.8% and 6.7%, for mirabegron and vibegron, respectively. (G) Emotions: not achieved, 24.3% and 33.3%; achieved, 75.7% and 66.7%, for mirabegron and vibegron, respectively. (H) Sleep/energy: not achieved, 43.2% and 51.3%; achieved, 56.8% and 48.7%, for mirabegron and vibegron, respectively. (I) Severity: not achieved, 21.6% and 36.8%; achieved, 78.4% and 63.2%, for mirabegron and vibegron, respectively. A reduction of ≥5 points in total score was defined as an MCIC. KHQ, King's Health Questionnaire; MCIC, minimal clinically important change.

**FIGURE 6:**
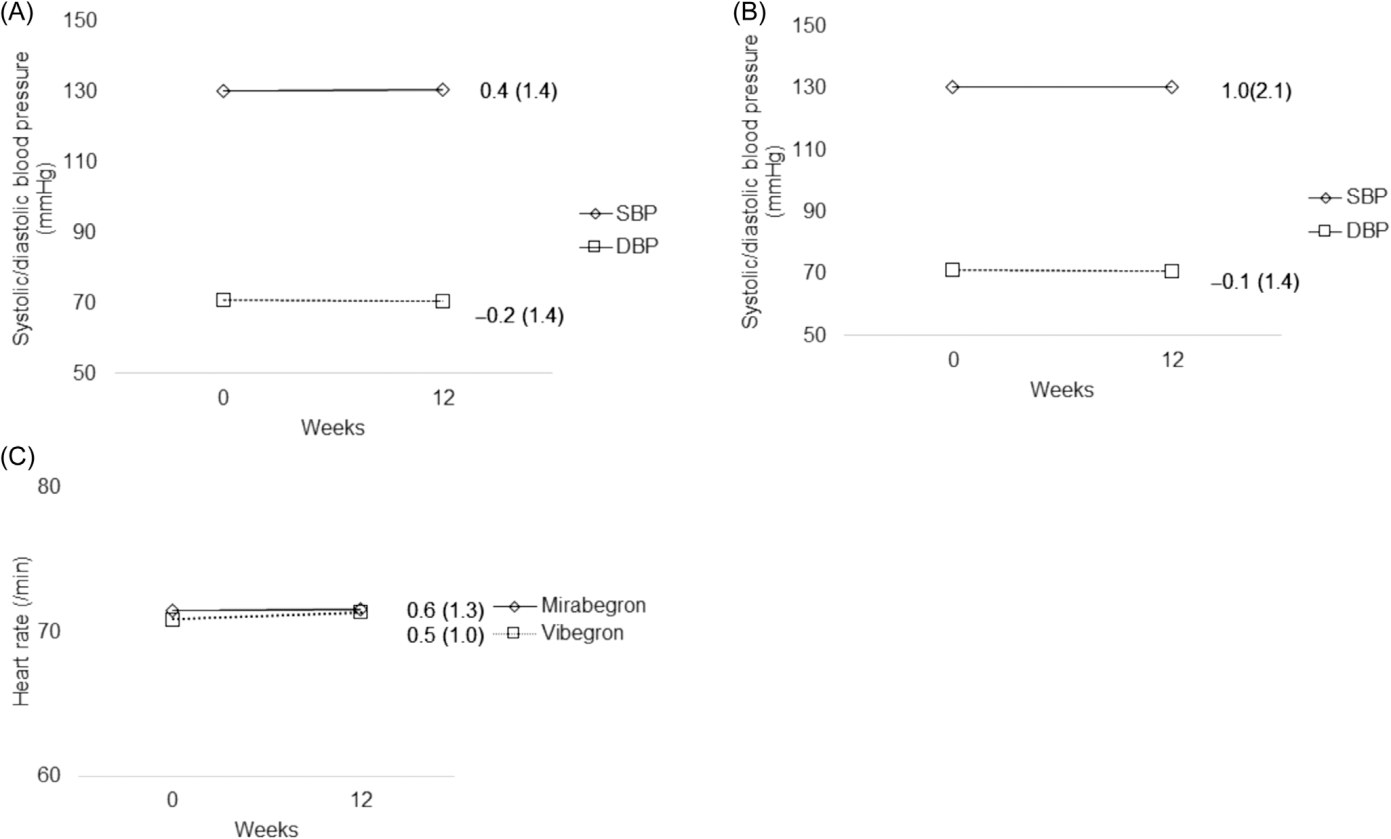
(A) Mean change in SBP and DBP in the mirabegron group. (B) Mean change in SBP and DBP in the vibegron group. (C) Mean change in heart rate in both groups. Data are presented as mean (SD). DBP, diastolic blood pressure; SBP, systolic blood pressure.

There was no significant difference between the mirabegron and vibegron groups in the proportion of patients who achieved a reduction of ≥3 points in MCIC of OABSS (64.9% vs. 84.2%; OR: 2.30; 95% CI: 0.78, 6.77), OAB dry (73.0% vs. 63.2%; OR: 0.70; 95% CI: 0.26, 1.89), or 5‐point reduction in each KHQ domain, namely, general health perceptions (48.6% vs. 21.1%; OR: 0.34; 95% CI: 0.12, 0.95), incontinence impact (59.5% vs. 63.2%; OR: 1.084; 95% CI: 0.43, 2.77), role limitations (54.1% vs. 65.8%; OR: 1.43; 95% CI: 0.56, 3.63), physical limitations (56.8% vs. 63.2%; OR: 1.20; 95% CI: 0.47, 3.09), social limitations (45.9% vs. 52.6%; OR: 1.17; 95% CI: 0.46, 2.99), personal relationships (14.8% vs. 6.7%; OR: 0.64; 95% CI: 0.17, 2.42), emotions (75.7% vs. 66.7%; OR: 0.75; 95% CI: 0.28, 2.06), sleep/energy (56.8% vs. 48.7%; OR: 0.77; 95% CI: 0.31, 1.90), and severity (78.4% vs. 63.2%; OR: 0.50; 95% CI: 0.17, 1.44) (Figures 4A,B, 5A–I, 6A–C, respectively; Table 2) at EOT.

The data on TRAEs are presented in Table S1. The overall incidence of TRAEs was higher in the vibegron group (38.5%) than in the mirabegron group (19.1%) (*p* = .047). The incidence of dry mouth was higher in the vibegron group (15.4%) than in the mirabegron group (12.8%), although this difference was not statistically significant. Similarly, the incidence of constipation was higher in the vibegron group (11.5%) than in the mirabegron group (2.1%). Serious TRAEs were observed in one patient (2.1%) in the mirabegron group and five patients (9.8%) in the vibegron group. The study drug was discontinued due to TRAEs, including gastritis in one patient in the mirabegron group and dizziness, dyspnea, cramps, arthralgia, palpitations, and urinary retention in five patients in the vibegron group. Further, one patient in the vibegron group developed dyspnea and palpitations. The onset of symptoms occurred within 1 week of administration in all patients. No statistically significant adjusted mean change in PVR from baseline to EOT was observed in either group (difference [SE]: 0.35 [17.2]; 95% CI: −34.3, 35.0) (Figure 3D; Table 2). Further, there were no significant changes in vital signs at baseline and EOT for both groups (Figure 6). In the mirabegron group, mean changes (SD) in parameters were as follows: SBP, 0.45 (1.4); DBP, −0.26 (1.4); and HR, 0.57 (1.3). In the vibegron group, mean changes (SD) in parameters were as follows: SBP, 1.1 (2.2); DBP, −0.19 (1.5); and HR, 0.46 (1.1). There were no significant differences in SBP, DBP, and HR between the two groups.

## 4 DISCUSSION

This is the first head‐to‐head prospective study to demonstrate the clinical characteristics and safety profiles of mirabegron and vibegron in patients with OAB. Overall, the results showed that there was no statistically significant difference between mirabegron and vibegron in terms of the change in OABSS from baseline to EOT. However, although the percentage of patients reaching MCIC on the OABSS from baseline to EOT was not statistically significant, we nevertheless believe that it is important from a clinical standpoint.

Previous studies have shown changes in the OABSS from baseline to EOT. A recent post hoc analysis involving 50 mg mirabegron reported that the change (mean ± SD) in the OABSS from baseline to EOT was −3.6 ± 3.22 for male and female patients <75 years.^24^ Another RCT reported that the change in OABSS was −4.51 ± 3.05 (mean age 73.5 years).^25^ A recent prospective study using 50 mg vibegron showed that the mean change in OABSS from baseline to EOT was −4.98 for men and women (mean age 73 years).^26^ The present study showed that treatment with both mirabegron and vibegron greatly exceeded the MCIC. The higher efficacy observed in this study may have been because the target population was female patients with no prior history of OAB.^27^, ^28^

In this study, age was adjusted as a covariate, and logistic analysis was performed to compare each of the nine domains of the KHQ. General health perceptions were significantly improved in the mirabegron group compared with those in the vibegron group, but there were no significant differences between the two groups in other domains. A previous RCT showed that KHQ domains, other than general health perceptions and personal relationships, were significantly improved in those who received 50 mg mirabegron^17^ and 50 mg vibegron^19^ compared to those in the participants who received the placebo. In this study, general health perceptions significantly improved in the mirabegron group compared with those in the vibegron group, but the results did not take multiplicity into account. Previous studies^17^, ^19^ have shown no cases of improvement in general health perceptions, making it difficult to explain whether this is a clinically important difference. Further research is needed to determine whether the improvement in QoL in clinical trials would differ between the two β_3_‐AR agonists in a real‐world setting.

Overall, this study showed that the 12‐week discontinuation rate due to TRAEs was lower in the mirabegron group than in the vibegron group. The incidence of dry mouth and constipation was lower in the mirabegron group than in the vibegron group. In particular, the presence of serious TRAEs, such as urinary retention, arthralgia, cramps, and dyspnea, also affected drug tolerability. A systematic literature review of 44 RCTs showed that 50 mg mirabegron produced a low rate of dry mouth and constipation, which are the most common AEs resulting in treatment discontinuation with most antimuscarinics.^29^ Further, the previous RCT reported that dry mouth and constipation were the most common TRAEs for vibegron, but the incidence of dry mouth was similar to that of placebo.^19^ A phase 3 RCT found that vibegron was well tolerated, with a 12‐week discontinuation rate of 1.7% compared to 1.1% for placebo.^30^ Overall, the AEs observed in the vibegron group in the present study contrasted with our expectations and could not be explained biologically. This remains an unresolved issue that requires further research.

This study has several limitations. First, because two β_3_‐AR agonists may be used for longer than 12 weeks, the 12‐week treatment period was too short to assess the actual efficacy and to generalize the long‐term efficacy, safety, and tolerability of the drug. Second, the sample size was relatively small. Third, the study was conducted at a single institution. Fourth, because this was an open‐label trial rather than a placebo‐controlled trial, there may be a risk of observer bias. Further, there were no cases of cardiovascular AEs specific to β_3_ agonists because the number of patients was not large enough to perform a SAF. We believe that continued attention should be paid to cardiovascular AEs in order to administer β_3_ agonists.

In summary, this was a study of Japanese treatment‐naïve postmenopausal female patients with OAB. Our analysis showed that 12 weeks of treatment with 50 mg mirabegron and 50 mg vibegron once daily both improved OAB symptoms, with no significant difference between the two drugs. Safety assessment showed a higher rate of more serious AEs leading to discontinuation in the vibegron group. A more detailed analysis of the efficacy and safety of the two β_3_‐AR agonists should be conducted.

## CONFLICT OF INTEREST STATEMENT

The authors declare no conflict of interest.

## Supporting information


**TABLE S1.** Details the treatment‐related adverse events (safety analysis).

## Data Availability

The data that support the findings of this study are available from the corresponding author upon reasonable request.
