# Supplementary material for: Mirabegron versus vibegron in previously untreated female patients with overactive bladder: A randomized, single‐clinic, open‐label trial
Source: Low Urin Tract Symptoms. 2023 May 4;15(4):129–38. doi: 10.1111/luts.12480 (PMC11500682; doi:10.1111/luts.12480)
Supplement: Supplementary file 1 — TABLE S1. Details the treatment‐related adverse events (safety analysis). [file LUTS-15-129-s001.pdf]

SuppInfo Table 1 Treatment-related adverse events (safety analysis)

Adverse event	Treatment group		
	Mirabegron: *n* = 47	Vibegron: *n* = 52	*P*
Number of patients	9	18	
Total percentage, *n* (%)	9 (19.1)	20 (38.5)	0.047*
Dry mouth	6 (12.8)	8 (15.4)	0.78
Constipation	1 (2.1)	6 (11.5)	0.12
Arthralgia	0	1 (1.9)	1.0
Urinary retention	0	1 (1.9)	1.0
Gastritis	2 (4.3)	0	0.22
Cramp	0	1 (1.9)	1.0
Dyspnea	0	1 (1.9)	1.0
Dizziness	0	1 (1.9)	1.0
Palpitations	0	1 (1.9)	1.0
